# A prognostic index for multiple myeloma.

**DOI:** 10.1038/bjc.1996.212

**Published:** 1996-05

**Authors:** G. Grignani, P. G. Gobbi, R. Formisano, C. Pieresca, G. Ucci, S. Brugnatelli, A. Riccardi, E. Ascari

**Affiliations:** Università di Pavia, IRCCS Policlinico S. Matteo, Italy.

## Abstract

The current prognostic systems have failed to identify multiple myeloma (MM) patients who require aggressive therapy. These staging systems do not reliably distinguish patients with different prognoses. This paper explores the possibility of improving the prognostic forecast in MM by considering some clinical characteristics at diagnosis together with response to first-line chemotherapy. A total of 231 patients were prospectively randomised in a multicentre trial to no therapy vs melphalan + prednisone (MP) for stage I, MP in stage II, and MP vs peptichemio, vincristine and prednisone for stage III. The clinical features of these groups were evaluated for prognostic variables predictive of overall survival by means of univariate and multivariate analysis. The independently significant variables were incorporated into a model that identified three groups of patients with different risks of death and different overall survival. Three variables retained statistical significance: the staging system proposed by the British Medical Research Council, a composite parameter integrating the percentage of bone marrow plasma cells with cytological features of the infiltrating elements (plasma cell vs plasmablast), and response to 6 months of first-line chemotherapy. These three variables led the proposal of a scoring system able to identify three different risk classes (with median overall survival of 52, 28 and 13 months respectively) and to estimate individual patient prognosis more flexibly. The proposed risk classes, drawn from both diagnostic and therapeutic parameters, are thought to be a clinical and investigational instrument for separating MM patients into comparable groups, for selecting the best available therapy and for evaluating response with respect to the disease of each new patient.


**British Journal of Cancer (1996) 73, 1101–1107**
© 1996 Stockton Press All rights reserved 0007–0920/96 $12.00

### A prognostic index for multiple myeloma

G Grignani, PG Gobbi, R Formisano, C Pieresca, G Ucci, S Brugnatelli, A Riccardi and E Ascari

*Medicina Interna e Oncologia Medica, Università di Pavia, IRCCS Policlinico S. Matteo, Pavia, Italy.*

**Summary** The current prognostic systems have failed to identify multiple myeloma (MM) patients who require aggressive therapy. These staging systems do not reliably distinguish patients with different prognoses. This paper explores the possibility of improving the prognostic forecast in MM by considering some clinical characteristics at diagnosis together with response to first-line chemotherapy. A total of 231 patients were prospectively randomised in a multicentre trial to no therapy *vs* melphalan + prednisone (MP) for stage I, MP in stage II, and MP *vs* peptichemio, vincristine and prednisone for stage III. The clinical features of these groups were evaluated for prognostic variables predictive of overall survival by means of univariate and multivariate analysis. The independently significant variables were incorporated into a model that identified three groups of patients with different risks of death and different overall survival. Three variables retained statistical significance: the staging system proposed by the British Medical Research Council, a composite parameter integrating the percentage of bone marrow plasma cells with cytological features of the infiltrating elements (plasma cell *vs* plasmablast), and response to 6 months of first-line chemotherapy. These three variables led the proposal of a scoring system able to identify three different risk classes (with median overall survival of 52, 28 and 13 months respectively) and to estimate individual patient prognosis more flexibly. The proposed risk classes, drawn from both diagnostic and therapeutic parameters, are thought to be a clinical and investigational instrument for separating MM patients into comparable groups, for selecting the best available therapy and for evaluating response with respect to the disease of each new patient.

**Keywords:** multiple myeloma; histopathology; prognosis; response to treatment; staging system

The survival duration of patients affected by multiple myeloma (MM) has not varied significantly in the last 20 years (Alexanian and Dimopoulos, 1994). In spite of this rather unsatisfactory situation, many current standard regimens seem to have improved patient quality of life. The results of myeloablative therapies followed by either autologous or allogeneic bone marrow transplantation (BMT) are promising but still burdened by uncertain outcomes. These approaches require a more accurate selection of the patients who would really benefit from such treatments. At the same time, the currently available prognostic criteria have never confirmed their actual predictive ability in selecting subsets of MM patients for different therapies.

There is considerable heterogeneity among the patient series reported in the literature, but reliable comparisons are only possible if patients are divided into well-defined and reproducible groups. As our current staging systems are not able to discriminate MM patients with different prognoses, the results of newer therapies are destined to remain controversial. Therefore we need more dependable instruments to establish a basis for more reliable comparability. Various authors have recognised this need (Niesvzky *et al*., 1993; Greipp, 1992).

In order to overcome the present impasse involving MM prognosis, we explored the possibility of integrating response to first-line treatment (TR) together with one of the three most common staging systems and other well-known clinical features at diagnosis. TR proved to be a major prognostic factor and allowed us to propose a new prognostic system for MM patients.

Correspondence: PG Gobbi, Medicina Interna e Oncologia Medica, Università di Pavia, IRCCS Policlinico S. Matteo, P.le Golgi n° 3, 27100 Pavia, Italy
Received 18 July 1995; revised 16 November 1995; accepted 17 November 1995

### Materials and methods

### *Study population*

Between 1987 and 1989, 231 MM patients staged according to Durie and Salmon (1975) were prospectively randomised in a multicentre trial to the following therapies: no therapy *vs* melphalan + prednisone (MP) for stage I patients, MP in stage II and MP *vs* peptichemio, vincristine and prednisone (PTC) (Riccardi *et al*., 1986) in stage III subjects. Patient characteristics are described in Table I,

Patients were evaluated for response at the end of the first 6 months of induction therapy according to slightly modified

**Table I** Characteristics of the 231 evaluable patients

| | |
|---|---|
| Mean age = 65 | |
| Male = 120 | |
| Female = 111 | |
| Isotype | |
| | IgG = 132 |
| | IgA = 55 |
| | IgD = 0 |
| | IgE = 1 |
| | $\kappa/\lambda = 43$ |
| *Stages* | |
| DS | |
| | I = 44 |
| | II = 101 |
| | III = 86 |
| MWJ | |
| | I = 92 |
| | II = 68 |
| | III = 71 |
| BMRC | |
| | I = 78 |
| | II = 124 |
| | III = 29 |

DS, Durie and Salmon; MWJ, Merlini–Waldenström–Jayakar; BMRC, British Medical Research Council.

clinical criteria adopted by the Southern Cancer Study Group (Cohen et al., 1986). Criteria were as follows: (a) reduction in the monoclonal component (MC); (b) decrease in bone marrow plasma cells (BMPC) of at least 20% or a return to less than 20% as evaluated on bone marrow imprints before and after treatment; (c) a $\geqslant 2$ g dl$^{-1}$ rise in haemoglobin (Hb) concentration in anaemic patients (Hb < 11 g dl$^{-1}$) sustained for more than 4 months; (d) return of serum calcium and blood urea nitrogen (BUN) to normal values; (e) elevation of serum albumin to 3 g dl$^{-1}$ or higher in the absence of other causes of hypoalbuminaemia; (f) absence of progression of skeletal lytic lesions.

Complete response (CR) was defined as a >50% reduction in the MC and a response in more than half of the other parameters. Partial response (PR) was a 25–50% reduction in the MC and a response in more than half of the other parameters. No response (NR) was defined as failure to fulfil the above criteria for CR and PR. Progression was a >25% increase in the MC and/or an increase in BMPC of at least 20% and/or a worsening of laboratory parameters (mainly haemoglobin, serum calcium, BUN) and/or skeletal lytic lesions.

The median survival of patients in stage I was 58 months (no therapy) vs 54 months (MP) ($P = 0.4701$). The median survival of patients in stage III was 31 months (MP) vs 34 months (PTC) ($P = 0.1274$). The mean follow-up was 42 months. Furthermore, there were no statistically significant differences between complete remission plus partial remission vs no remission plus progression among the compared groups. So we can conclude that the prognosis of our study population was not heavily or differentially influenced by therapy.

*Statistical analysis*

The following clinical and laboratory parameters were measured at diagnosis and evaluated for prognostic relevance: age, sex, haemoglobin (g dl$^{-1}$), white blood cell count ($\times 10^9$ l$^{-1}$), platelets ($\times 10^9$ l$^{-1}$), serum creatinine (mg dl$^{-1}$), BUN (mg dl$^{-1}$), calcium (mg dl$^{-1}$), serum albumin (g dl$^{-1}$), MC (g dl$^{-1}$), MC isotype, Bence-Jones proteinuria, hydroxyprolinuria (mg dl$^{-1}$), serum alkaline phosphatase (mU ml$^{-1}$), erythrocyte sedimentation rate (ESR; mm/first hour), serum $\beta_2$-microglobulin ($\beta_2$M; $\mu$g ml$^{-1}$), performance status (according to the Karnofsky index scale), number of lytic bone lesions, clinical stage according to three different staging systems [Durie and Salmon, DS (Durie and Salmon, 1975); Merlini–Waldenström–Jayakar, MWJ (Merlini et al., 1980); British Medical Research Council (Medical Research Council's Working Party on Leukemia in Adults, 1980), BMRC], bone marrow plasma cell percentage (BMI), bone marrow plasma cell cytological feature (plasma cell vs plasmablast) (Bartl et al., 1982) (BMC), bone marrow infiltrate pattern (interstitial vs diffuse), osteoclastic activity (low, intermediate, high), bone marrow fibrosis (low, intermediate, high), bone marrow cellularity (ratio between nucleate population and adipose tissue, graded as hypoplastic, normal or hyperplastic).

Lastly, we studied the impact of TR on prognosis. This unusual approach is supported by the following facts: (a) many standard-dose combination chemotherapies were compared with melphalan and prednisone (MP) but none was shown to be superior (Gregory et al., 1992; Cooper et al., 1986; MacLennan et al., 1988; Pavlovsky et al., 1988; Tribalto et al., 1985) and thus MP still represents the reference first-line chemotherapy; (b) response to conventional chemotherapy is the most powerful prognostic factor for patients undergoing either allogeneic (Gahrton et al., 1993) or autologous BMT (Jagannath et al., 1990; Dimopoulos et al., 1993; Fernand et al., 1993); (c) there is a broad consensus among experts (Cunningham et al., 1994) that the outcome of first-line therapy is the guideline for deciding further treatment because unresponsive patients are unlikely to benefit, in terms of survival, from additional treatment; (d) as a matter of fact, response to 6 months of treatment with either an alkylating agent plus prednisone or combination chemotherapy was recently confirmed as a very useful prognostic factor. (Guillemin et al., 1995). TR was categorised as 0 for complete or partial remission, and as 1 for progression or stable disease.

Overall survival was computed according to Kaplan and Meier (Kaplan and Meier, 1958). All deaths were considered as events regardless of their cause. Each patient was considered to be alive at the time of his/her last evaluation unless death had been documented. Differences in overall survival between groups were analysed using the log-rank test and taking censored data into account.

Multivariate analysis of survival was performed using a step-up selection procedure (Armitage and Berry, 1987) and the Cox proportional hazards model (Cox, 1972). We checked the goodness of fit of the selected models by means of the Akaike information criterion (AIC) (Akaike, 1974). Briefly, if $lm$ is the log-likelihood of model $m$ and $dfm$ is the degree of freedom of model $m$, then $AIC = 2\ lm + 2\ dfm$. The best model is the one with the lowest AIC value.

### Results

The following prognostic variables were statistically significant ($P < 0.05$) at univariate analysis: age, sex, haemoglobin, serum creatinine, BUN, serum calcium, serum albumin, amount of the MC, Bence-Jones protein, hydroxyprolinuria, serum alkaline phosphatase, ESR, $\beta_2$M, Karnofsky performance status, number of bone lytic bone lesions, stage according to each of the three clinical systems, BMI, BMC, bone marrow infiltrate pattern, bone marrow cellularity and TR (Table II).

The log-likelihoods of the three staging systems, BMRC, DS and MWJ, were not statistically different; nevertheless BMRC staging showed the best log-likelihood and thus we decided to adopt this system because of its relative simplicity and greater reproducibility.

**Table II** Single-parameter analysis of prognostic variables

| *Parameter* | P-*value* |
|---|---|
| Age (years) | 0.0016 |
| Sex | 0.0134 |
| Haemoglobin (g l$^{-1}$) | 0.0000 |
| White blood cell ($\times 10^9$ l$^{-1}$) | 0.1849 |
| Platelets ($\times 10^9$ l$^{-1}$) | 0.2182 |
| Serum creatinine (mg dl$^{-1}$) | 0.0164 |
| Blood urea nitrogen (mg dl$^{-1}$) | 0.0001 |
| Serum calcium (mg dl$^{-1}$) | 0.0098 |
| Serum albumin (g dl$^{-1}$) | 0.0002 |
| Monoclonal component (g dl$^{-1}$) | 0.0043 |
| Monoclonal component isotype | 0.0673 |
| Bence-Jones protein (+/–) | 0.0001 |
| Hydroxyprolinuria (mg dl$^{-1}$) | 0.0062 |
| Serum alkaline phosphatase (mU ml$^{-1}$) | 0.0189 |
| Erythrocyte sedimentation rate (mm/first hour) | 0.0001 |
| Serum $\beta_2$-microglobulin ($\mu$g ml$^{-1}$) | 0.0001 |
| Karnofsky performance index | 0.0002 |
| Number of lytic bone lesions | 0.0001 |
| Stage Durie–Salmon | 0.0000 |
| Stage Merlin–Waldenström–Jayakar | 0.0000 |
| Stage British Medical Research Council | 0.0000 |
| Bone marrow plasma cell percentage | 0.0001 |
| Bone marrow infiltrate cytology (plasma cell/plasmablastic) | 0.0001 |
| Bone marrow infiltrate pattern (interstitial/diffuse) | 0.0006 |
| Bone marrow fibrosis (low/intermediate/high) | 0.0814 |
| Bone marrow cellularity (nucleate population/adipose tissue) | 0.0561 |
| Osteoclastic activity (low/intermediate/high) | 0.1876 |
| Response to therapy | 0.0000 |

**Table III** Multivariate analysis of significant variables

| *Parameter* | *Log-likelihood* | P-*value* |
|---|---|---|
| Stage British Medical Research Council | −649.230 | 0.0043 |
| Bone marrow plasma cell percentage | −643.664 | 0.0178 |
| Bone marrow infiltrate cytology (plasma cell/plasmablastic) | −640.356 | 0.0193 |
| Bone marrow percentage and cytology of infiltrate (BMIC) | −640.389 | 0.0198 |
| Response to therapy (CR+PR *vs* progression) | −628.146 | 0.0001 |

Since there were too many prognostic variables with respect to the number of cases examined, we followed a step-up procedure in our regression analysis. In other words, we began our multivariate analysis by adding each single parameter to BMRC stage.

All histopathological parameters examined were statistically significant at this first step of our procedure. We then looked at whether any one of them provided more prognostic information than the others. Two–BMI and BMC–were found to be the only statistically significant ones in a multivariate analysis of all the histopathological variables. BMI was dichotomised according to its best cut-off value of 40% bone marrow infiltration and then combined with BMC into a single variable called bone marrow infiltrate and cytological type (BMIC), defined as follows: 0 if both are favourable; 1 if either is unfavourable; 2 if both variables are unfavourable.

Afterwards we repeated the analysis with the remaining significant variables and found that only three of them retained their importance: two characteristics at diagnosis, BMRC stage and BMIC, and the 'ongoing' variable TR. A model containing these three prognostic factors was then constructed and it proved to be highly statistically significant (Table III). Moreover, this model confirmed its validity when tested by means of the Akaike information criterion (Table IV).

Since the relative risks associated with each of the independently significant prognostic variables were comparable (Table V), the relative risk of death for a patient could be characterised by adding the number of risk factors. We propose a simple scoring system ranging from 0 to 5, according to the combination of risk factors present. This prognostic scoring is based on the patient's prognostic variables at diagnosis–BMRC stage and BMIC–and his/her TR evaluated after a 6 month period of conventional chemotherapy, and is defined in this way: 0 for stage I, positive response to treatment and favourable BMIC; 1 for stage II, an intermediate BMIC and progression after first-line treatment; 2 for stage III and unfavourable BMIC.

Scoring from 0 to 5 allows us to stratify patients into three different classes of increasing risk: class I from 0 to 1; class II equal to 2; class III from 3 to 5. The three classes have remarkably dissimilar overall survival curves with highly statistically significant differences ($P<0.001$) (Figure 1). The median survival and death percentages per class are shown in Table VI.

The Cox proportional hazards model was applied–as an alternative analysis–to verify the results achieved by the scoring system. The values of the $\beta$ coefficients of the Cox model are shown in Table VII. It is well known that these coefficients are an estimate of the relative weight of each covariate (i.e. each specific prognostic factor). Therefore the $\beta$s can be used to produce and evaluate another prognostic index, called the MM index, which is derived by summing the products of the value of each prognostic factor for the corresponding $\beta$ coefficient. The formula for this index is:

MM index = 0.5 × BMRC (i.e. 1 or 2 or 3) + 0.5 × BMIC (i.e. 1 or 2 or 3) + 1.5 × TR (i.e. 1 or 2)

Thus a patient with a II BMRC stage, 60% bone marrow involvement by a plasmablastic infiltrate and a poor response to therapy would have an index of 5.5 (0.5 × 2 + 0.5 × 3 + 1.5 × 2). This index, which ranges from 2.5 to 6, allows a stratification that substantially confirms the results obtained with the scoring system. The first group of patients presents an index from 2.5 to 3; the second group from 3.5 to 4; the third group of patients has an index of 4.5 or more. These three groups also show dissimilar overall survivals with highly statistically significant differences (Figure 2).

**Table IV** Akaike information criterion (AIC) (Akaike, 1974) of the best prognostic indicators

| *Prognostic indicators* | *Log-likelihood* | *AIC* |
|---|---|---|
| DS | 615.669 | 1234.502 |
| MWJ | 616.147 | 1239.556 |
| BMRC | 610.669 | 1225.338 |
| BMIC | 611.362 | 1226.724 |
| TR | 613.794 | 1229.588 |
| BMRC+BMIC | 601.779 | 1223.558 |
| BMRC+TR | 601.035 | 1208.070 |
| BMIC+TR | 601.008 | 1208.016 |
| BMRC+BMIC+TR | 592.559 | 1192.118 |

**Table V** Relative risks of prognostic factors

| *Parameter* | *Relative risk* | P-*value* |
|---|---|---|
| British Medical Research Council | | |
| Stage I *vs* stage II | 3.34 | <0.001 |
| Stage II *vs* stage III | 3.41 | <0.001 |
| BMIC | | |
| Favourable *vs* intermediate | 2.94 | <0.001 |
| Intermediate *vs* unfavourable | 2.97 | <0.001 |
| Response to treatment | | |
| (CR+PR) *vs* (progression+NR) | 3.26 | <0.001 |

**Figure 1** Overall survival according to risk class.

### Discussion

The survival of patients with MM has not changed substantially in the last 25 years. Thus, within a median disease survival of about 3 years we find patients who live for

10 years or even longer and others who die in less than 1 year. The search for a better definition of MM prognosis has led to the development of many different staging systems and the use of various prognostic factors. Most of them, while promising, have failed to identify homogeneous and reproducible risk classes of MM patients able to orient treatment. It is therefore not surprising that recently (Alexanian and Dimopoulos, 1994; Kyle, 1993) no criterion was proposed other than the absence of symptoms for deciding whether or not to treat a newly diagnosed MM, regrdless of stage, labelling index, $\beta_2$M, serum thymidine kinase or any other clinical feature.

The lack of improvement in survival is mainly due to the poor efficacy of our current therapies, both conventional and myeloablative followed by stem cell rescue (regardless of whether the source of the stem cell is the bone marrow–allogeneic or autologous–or the peripheral blood). Nevertheless, the only long survivors are transplanted patients and most of our efforts to cure MM rely upon some form of transplantation (Kyle, 1993; Gahrton *et al.*, 1991). It is thus of great importance to avoid the risk of useless delays in identifying a good candidate for this procedure.

None of the present prognostic instruments are reliable enough to identify which patients should receive transplantation. One clinical and rather popular criterion is responsiveness to first-line chemotherapy. Indeed, on the other hand, the probability of developing a multiresistant neoplastic clone is enhanced by merely selective antineoplastic drug pressure and, on the other hand, an early resistant MM patient will probably not reach a complete remission even with myeloablative regimens (Harousseau *et al.*, 1992). Furthermore, experimental evidence demonstrates reduced mobilisation of peripheral blood stem cells in heavily pretreated patients (Kotasek *et al.*, 1992). As the peripheral blood stem cell transplantation procedure seems to be the most promising and widely appliable approach, early patient selection could enhance the probability of success.

The aim of this work was to propose an improved tool better able to stratify MM patients as soon as possible in order to treat them with the best available therapy for their disease. The following considerations illustrate the reasons supporting our choice of prognostic variables and the proposed risk classes or prognostic index.

### *BMRC stage*

Among the many different proposed staging systems, we studied three: DS, MWJ and BMRC. Each has its own peculiarities resulting from the point of view from which it explores the disease. Unfortunately, we had incomplete data about C reactive protein so that we could not evaluate the impact of a fourth staging system, that of Bataille (1992). However, our multivariate analysis did not show an independent role for $\beta_2$M, which is the second parameter of Bataille's system, when included in the analysis with the three ultimately selected factors. This result surprised us because of the general consensus regarding the prognostic importance of $\beta_2$M. Nevertheless, some authors did not find $\beta_2$M to be significant (Fernand *et al.*, 1993; Peest *et al.*, 1993; Cunningham *et al.*, 1994; Bladé 1993). We will briefly discuss the three systems that were considered.

DS staging system evaluates the neoplastic mass with the assumption that the higher the number of plasma cells the poorer is prognosis. The MWJ staging system assumes an exponential survival distribution for MM patients. This property allows the authors to develope a parametric model in which the immunoglobulins produced – IgG, IgA or light chains $\kappa$ and $\lambda$ – are important prognostic factors. The BMRC staging system considers the presence of symptoms and 2 biochemical parameters: blood nitrogen and haemoglobin concentrations.

In our analysis all the staging systems considered were statistically significant at both univariate and multivariate analysis. We chose the BMRC system for the following reasons: (a) simplicity, (b) importance given to symptoms, (c) best improvement in the log-likelihood in our multivariate analysis by means of the Akaike information criterion. Furthermore, some of us had already demonstrated the relative superiority of BMRC over both DS and MWJ (Gobbi *et al.*, 1991).

The BMRC staging system describes the prognosis well in our series, too. A major weakness of this staging system is that it identifies too many patients as stage II. In our data the distribution according to the BMRC staging system was: 30% (stage I); 55% (stage II); 15% (stage III). Our proposed risk classes are sharper and separate BMRC stage II into three almost equal classes (Table VIII). Table VIII also shows how the survival and percentages of death among the redistributed BMRC stage II patients are different.

### *BMIC*

Many aspects of the cellular biology of MM escape our comprehension. In particular, we do not know what the proliferating cell is or where the MM stem cell comes from. An understanding of these two questions would enhance our grasp of the biology and clinical behaviour of this neoplasia.

**Table VI** Risk classes

| *Risk class* | *Score* | *Median survival (months)* | P-*value* | *Death (%)* |
|---|---|---|---|---|
| Class I | 0–1 | 52 | | 59 |
| | | | 0.0001 | |
| Class II | 2 | 28 | | 82 |
| | | | 0.0001 | |
| Class III | 3–5 | 13 | | 98 |

**Figure 2** Overall survival according to MM index.

**Table VII** Cox proportional hazards model

| *Parameter* | $\beta$ | *s.e.* | *Exp-$\beta$* | P-*value* |
|---|---|---|---|---|
| Stage British Medical Research Council (BMRC) | 0.538191 | 0.1407727 | 1.712905 | 0.0013 |
| Bone marrow percentage and cytology of infiltrate (BMIC) | 0.468012 | 0.1124041 | 1.596817 | 0.0025 |
| Response to therapy: (CR + PR) *vs* (progression + NR) (TR) | 1.336513 | 0.2351125 | 3.805751 | 0.0001 |

Table VIII Differences in distribution between the BMRC staging system and the risk classes

| Risk class | Median survival (months) | BMRC staging system | Death in BMRC stage (%) |
|---|---|---|---|
| Class I 100 patients | 52 | I = 60 | 57 |
| | | II = 40 | 62 |
| Class II 66 patients | 28 | I = 13 | 79 |
| | | II = 44 | 82 |
| | | III = 9 | 84 |
| Class III 65 patients | 13 | I = 7 | 89 |
| | | II = 40 | 94 |
| | | III = 18 | 99 |

Nevertheless, it seems at least reasonable, as occurs for many other tumours, that biology somehow correlates with the tumour mass at diagnosis and with the characteristics of the cells making up the mass itself. Since the first paper by Bartl *et al.* (1982) MM histology and cytopathology have been considered by many authors to be relevant prognostic factors (Greipp *et al.*, 1985; Bartl, 1988; Moro *et al.*, 1992; Peest *et al.*, 1993; Sukpanichnant *et al.*, 1994). In the present work we considered many histopathological aspects, but only the percentage of bone marrow infiltration by plasma cells and the cytological features of this infiltrate retained statistical significance in a multivariate model. Furthermore, we were able to combine the 2 into 1 variable that expresses all the prognostic variability owing to MM histopathology.

It is evident that a higher BMI means a reduced environment for the normal components of the haemato-poietic system. In addition, the kinetics of the MM clone is slower than that of the normal haemopoietic cell lines (Drewinko *et al.*, 1981). This means that a higher percentage of infiltration will generally be due to a longer presence of the neoplasia. It is reasonable to think that the duration of the disease roughly correlates with the damage produced by the disease, with the number of cell duplications, and the risk of cell mutation in the direction of drug resistance [according to the so-called Goldie and Coldman hypothesis (Goldie and Coldman, 1979)].

The grade of immaturity of the neoplastic cells is a common oncological criterion for staging a tumour. This is particularly true in haematology, as shown by the various lymphoma or leukaemia classifications. The following experimental evidence (Kubagawa *et al.*, 1979; Pilarski *et al.*, 1985; Caligaris-Cappio *et al.*, 1985, 1992; Epstein *et al.*, 1990) supports the prognostic role of the prevailing type of cell: (a) plasma cells do not proliferate significantly; (b) in most cases we do not find circulating plasma cells in peripheral blood; (c) plasmablasts can circulate and, in the suitable microenvironment, start to proliferate, secrete Ig and differentiate to plasma cells. In other words, plasma cells are probably responsible only for the damage produced by the immunoglobulins secreted. Many authors (Fitz *et al.*, 1984; Carter *et al.*, 1987; San Miguel *et al.*, 1987; Paule *et al.*, 1988; Pasqualetti *et al.*, 1990; Greipp, 1992) have shown the survival advantage of plasmacytic *vs* plasmablastic type MM but this aspect has not been considered in the design and evaluation of clinical trials.

### *Response to treatment (TR)*

Considering TR as a prognostic variable may seem a tautology. It is self-evident that a responsive disease is more likely to have a better outcome than a non-responsive one. However, conventional chemotherapy does not have a major impact on survival in MM because it cannot eradicate the neoplastic clone. On the other hand, a response to therapy indicates a disease that is not yet fully resistant. Such a disease is still susceptible to cytoreduction with an appropriate therapy. In other words, we do not know the biological characteristics that make a cell 'resistant' to therapy, but TR is a good indirect marker of those cellular events that render a tumour composed mostly of cells either responsive or non-responsive. Other authors (Bugliosi *et al.*, 1994; Gahrton *et al.*, 1991; Jagannath *et al.*, 1990; Attal *et al.*, 1992; Gore *et al.*, 1989; Dimopoulos *et al.*, 1993; Fernand *et al.*, 1993; Johnson and Selby, 1994; Guillemin *et al.*, 1995) have considered TR as a prognostic factor because of its demonstrated importance on survival and on response to further therapies (Dimopoulos *et al.*, 1993; Fernand *et al.*, 1993). A recent report (Alexanian *et al.*, 1994) showed that myeloablative therapies are useless for MM patients in the following three conditions: resistant relapse, primary resistance longer than 1 year and relapse in consolidation therapy of a late remission.

There is one more reason supporting TR as a prognostic factor. MM is a neoplasm found in older people: the median age at diagnosis is 68 (Riccardi *et al.*, 1991; Longo, 1994). Thus most of these patients cannot stand aggressive chemotherapy. We still need an instrument to test the potential responsiveness of those who are considered candidates for myeloablative therapy. The efficacy of prior standard chemotherapy could represent a means for selecting those patients who deserve aggressive chemotherapy because they will hopefully be long survivors and eventually be cured.

### *Scoring system as a clinical tool*

We suggest that a direct clinical application of the proposed risk classes could be in choosing the therapeutical strategy for new patients. For example, the first class has a general outcome that is similar to the average outcomes of transplanted MM patients; however, the younger patients ($\leqslant$55 years) in this class might derive additional benefit from autologous transplantation – mostly from peripheral circulating progenitors (PCPs). The second class demonstrates a much poorer outcome. When feasible, transplantation can be attempted. In particular, depending on age and the availability of an HLA compatible donor, allogeneic transplantation seems to be appropriate; otherwise, if the patient is less than 65 years old autologous transplantation (preferentially from PCPs) can be considered. In our opinion, the third class is not curable with the current therapeutical strategies. Nevertheless, if the patient has a compatible donor and an acceptable age, allogeneic transplantation probably represents the only procedure able to offer some hope for this class. So far, the results in this subset of patients have been extremely poor.

The prognostic factors selected were used to construct two different scoring systems (risk classes and MM index) that ultimately corresponded to the same substantial prognostic index. While the scoring systems are derived from different approaches, the resulting prognostic index delineates three groups of patients with very similar overall survival, as shown in Figures 1 and 2. This is a relatively good confirmation that the two prognostic systems identify the same groups of patients with different risks.

For clinical use we have a slight preference for the risk classes because of their greater simplicity. Nevertheless, the MM index generated by the Cox analysis is equally sharp in separating the different prognostic groups, and it offers a matchless advantage for investigational uses. As a matter of fact, the MM index can be employed as a distinct prognostic covariate to be entered in a Cox's proportional hazards regression model applied to a therapeutic trial. Such an analysis would be simpler and easier. Most importantly, it would allow good accuracy in evaluating clinical results even in decidedly smaller samples. With this technique the MM index covariate would synthetically summarise the whole constellation of major and minor prognostic factors affecting the course of MM, so that a simple clinical trial would need no more than one other covariate: one expressing 'treatments', to test the null hypothesis (e.g. no differences between therapy A and B). Since the advisable number of covariates in a Cox's model should be no more than 5–10% of the number of events (deaths, null responses, relapses or others), an anlysis that evaluates the goodness of a treatment would be correct when 20–40 events have occurred. It is evident that even small institutions will be able to reach such a modest number of events and conclude a trial within a very few years. This would enhance clinical research on MM by lowering the number of patients necessary for individual clinical studies on the effectiveness of a drug or a treatment strategy.

Our approach is very empirical and clinically oriented, but it offers a feasible, reliable solution for better-tailored therapy until either new therapeutic devices or new biological markers are able to permit better stratification of MM patients at diagnosis.

**Acknowledgements**
Supported in part by a grant from the Ministero per la Ricerca Scientifica e Tecnologica, Rome, and the Fondazione 'Adolfo Ferrata e Edoardo Storti', Pavia.

### References

AKAIKE H. (1974). A new look at the statistical model identification. *IEEE Trans. Automat. Contr.*, **19,** 716–723.

ALEXANIAN R AND DIMOPOULOS M. (1994). The treatment of multiple myeloma. *N. Engl. J. Med.*, **330,** 484–489.

ALEXANIAN R, DIMOPOULOS M, SMITH T, DELASALLE K, BARLOGIE P AND CHAMPLIN R. (1994). Limited value of myeloablative therapy for late multiple myeloma. *Blood*, **83,** 512–516.

ARMITAGE P. AND BERRY G. (1987). *Statistical Methods in Medical Research*. Blackwell Scientific Publications: Oxford.

ATTAL M, HUGUET F, SCHLAIFER D, PAYEN C, LAROCHE M, FOURNIE B, MAZIERES B, PRIS J AND LAURENT G. (1992). Intensive combined chemotherapy for previously untreated aggressive myeloma. *Blood*, **79,** 1130–1136.

BARTL R. (1988). Histologic classification and staging of multiple myeloma. *Hematol. Oncol.*, **6,** 107–113.

BARTL R, FRISCH B, BURKHARDT R, FATEH-MOGHADAM A, MAHL G, GIERSTER P, SUND M AND KETTNER G. (1982). Bone marrow histology in myeloma: its importance in diagnosis, prognosis, classification and staging. *Br. J. Haematol.*, **51,** 361–375.

BATAILLE R, BOCCADORO M, KLEIN B, DURIE B AND PILERI A. (1992). C-reactive protein and b-2 microglobulin produce a simple and powerful myeloma staging system. *Blood*, **80,** 733–737.

BLADÈ J, SAN MIGUEL JF, ALCALÀ A, MALDONADO J, SANZ MA, GARCIA-CONDE J, MORO MJ, ALONSO C, BESALDUCH J, ZUBIZARRETA A, BESSES C, GONZALES-BRITO G, HERNANDEZ-MARTIN J, FERNADEZ-CALVO J, RUBIO D, ORTEGA F, JIMENEZ R, COLOMINAS P, FAURA MV, FONT L, TORTOSA J, DOMINGO A, FONTANILLAS M, ROZMAN C AND ESTAPÉ J. (1993). Alternating combination VCMP/VBAP chemotherapy versus Melphalan/Prednisone in the treatment of multiple myeloma: a randomized multicentric study of 487 patients. *J. Clin. Oncol.*, **11,** 1165–1171.

BUGLIOSI R, TRIBALTO M, AVVISATI G, BOCCADORO M, DE MARTINIS C, FRIERA R, MANDELLI F, PILERI A, PAPA G. (1994). Classification of patients affected by multiple myeloma using a neural network software. (letter) *Eur. J. Haematol.*, **52,** 182–183.

CALIGARIS-CAPPIO F, BERGUI L, TESIO L, PIZZOLO G, MALAVASI F, CHILOSI M, CAMPANA D, VAN CAMP B AND JANNOSSY G. (1985). Identification of malignant plasma cell precursors in the bone marrow of multiple myeloma. *J. Clin. Invest.*, **76,** 1243–1251.

CALIGARIS-CAPPIO F, GREGORETTI MG, GHIA P AND BERGUI L. (1992). In vitro growth of human multiple myeloma: implications for biology and therapy. *Hematol. Oncol. Clin. North. Am.*, **6,** 257–271.

CARTER A, HOCHERMAN I, LINN S, COHEN Y AND TATARSKY I. (1987). Prognostic significance of plasma cell morphology in multiple myeloma. *Cancer*, **60,** 1060–1065.

COHEN HJ, BARTOLUCCI AA, FORMAN WB AND SILBERMAN HR FOR THE SOUTHERN CANCER STUDY GROUP. (1986). Consolidation and maintenance therapy in multiple myeloma: randomized comparison of a new approach to therapy after initial response to treatment. *J. Clin. Oncol.*, **4,** 888–899.

COOPER MR, MCINTYRE OR, PROPERT KJ, PROPERT KJ, KOCHWA S, ANDERSON K, COLEMAN M, KYLE RA, PRAGER D, RAFLA S AND ZIMMER B. (1986). Single, sequential and multiple alkylating agent therapy for multiple myeloma: a Cancer and Leukemia Group B experience. *J. Clin. Oncol.*, **4,** 1331–1339.

COX D. (1972). Regression models and life-tables (with Discussion). *J. R. Stat. Soc.*, **34,** 187–220.

CUNNINGHAM D, PAZ-ARES L, MILAN S, POWLES R, NICOLSON M, HICKISH T, SELBY P, TRELEAVAN J, VINER C, MALPAS J, FINDLAY M, RAYMOND J AND GORE ME. (1994). High-dose melphalan and autologous bone marrow transplantation as consolidation in previously untreated myeloma. *J. Clin. Oncol.*, **12,** 759–763.

DIMOPOULOS MA, ALEXANIAN R, PRZEPIORKA D, HESTER J, ANDERSSON P, GIRALT S, MEHRA R, VAN BESIEN K, DELASALLE KB, READING C, DEISSEROTH AB AND CHAMPLIN RE. (1993). Thiotepa, busulfan and cyclophosphamide: a new preparative regimen for autologous marrow or blood stem cell transplantation in high-risk multiple myeloma. *Blood*, **82,** 2324–2328.

DREWINKO B, ALEXANIAN R, BOYER H, BARLOGIE B AND RUBINOW SI. (1981). The growth fraction of human myeloma. *Blood*, **57,** 333–338.

DURIE BGM AND SALMON SE. (1975). A clinical staging system for multiple myeloma. *Cancer*, **36,** 842–854.

EPSTEIN J, XIAO H AND HE XY. (1990). Markers of multiple haematopoietic-cell lineage in multiple myeloma. *N. Engl. J. Med.*, **322,** 664–668.

FERNAND JP, CHEVRET S, RAVAUD P, DIVINE M, LEBLOND V, DREYFUS F, MARIETTE X AND BROUET JC. (1993). High-dose chemoradiotherapy and autologous blood stem cell transplantation in multiple myeloma: results of a phase II trial involving 63 patients. *Blood*, **82,** 2005–2009.

FRITZ E, LUDWIG H AND KUNDI M. (1984). Prognostic relevance of cellular morphology in multiple myeloma. *Blood*, **63,** 1072–1079.

GAHRTON G, TURA S, LJUNGMAN P, BELANGER C, BRANDT L, CAVO M, FACON T, GRANENA A, GORE M, GRATWOHL A, LOWENBERG B, NIKOSKELAINEN J, REIFFERS JJ, SAMSON D, VERDONCK L AND VOLIN L. (1991). Allogeneic bone marrow transplantation in multiple myeloma. *N. Engl. J. Med.*, **325,** 176–179.

GOBBI PG, BERTOLONI D, GRIGNANI G, PIERESCA C, ROSSI A, RUTIGLIANO L, MERLINI G, RICCARDI A AND ASCARI E. (1991). A plea to overcome the concept of 'staging' and related inadequacy in multiple myeloma. *Eur. J. Haematol.*, **46,** 177–181.

GOLDIE JH AND COLDMAN AJ. (1979). A mathematical model for relating the drug sensitivity of tumors to the spontaneous mutation rate. *Cancer Treat. Rep.*, **63,** 1727–1733.

GORE ME, SELBY PJ, VINER C, CLARCK PI, MELDRUM M, MILLAR B, BELL J, MAITLAND JA, MILAN S, JUDSON IR, ZUIABLE A, TILLYER C, SLEVIN M, MALPAS JS AND MCELWAIN TJ. (1989). Intensive treatment of multiple myeloma and criteria for complete remission. *Lancet*, **2,** 879–882.

GREGORY WM, RICHARDS MA AND MALPAS JS. (1992). Combination chemotherapy versus melphalan and prednisone in the treatment of multiple myeloma: an overview of published trials. *J. Clin. Oncol.*, **10**, 334–342.

GREIPP PR. (1992). Advances in the diagnosis and management of myeloma. *Semin. Hematol.*, **29** (suppl. 2), 24–45.

GREIPP PR, RAYMOND NM, KYLE RA AND O'FALLON WM. (1985). Multiple myeloma: Significance of plasmablastic subtype in morphological classification. *Blood*, **65**, 305–310.

GUILLEMIN F, GUERCI A-P, FEUGIER P, PÉRÉ P, POUREL J AND GUERCI O. (1995). Development of a criterion for response to therapy at 6 months in multiple myeloma. *Eur. J. Haematol.*, **55**, 110–116.

HAROUSSEAU JL, MILPIED N, LAPORTE JP, COLLOMBAT P, FACON T, TIGAUD JD, CASASSUS P, GUILHOT F, IRFRAH N AND GANDHOUR C. (1992). Double-intensive therapy in high-risk multiple myeloma. *Blood*, **79**, 2827–2833.

JAGANNATH S, BARLOGIE B, DICKE K, ALEXANIAN R, CHESON B, LEMAISTRE FC, SMALLWOOD L, PRUITT K AND DIXON DO. (1990). Autologous bone marrow transplantation in multiple myeloma: identification of prognostic factors. *Blood*, **76**, 1860–1866.

JOHNSON PWM AND SELBY PJ. (1994). The treatment of multiple myeloma–an important MRC trial. *Br. J. Cancer*, **70**, 781–785.

KAPLAN EL AND MEIER P. (1958). Nonparametric estimation from incomplete observations. *J. Am. Stat. Assoc.*, **53**, 457–481.

KOTASEK D, SHPHERD KM, SAGE RE, DALE BM, NORMAN JE, CHARLES P, GREGG A, PILLOW A AND BOLTON A. (1992). Factors affecting blood stem cell collections following high-dose cyclophosphamide in lymphoma, myeloma and solid tumors. *Bone Marrow Transplant.*, **9**, 11–17.

KUBAGAWA H, VOGLER LB, CAPRA JD, CONRAD ME AND LAWTON AR. (1979). Studies of the clonal origin of multiple myeloma. *J. Exp. Med.*, **150**, 792–807.

KYLE RA. (1993). Newer approaches to the management of multiple myeloma. *Cancer*, **72**, 3489–3494.

LONGO DL. (1994). Plasma cell disorders. In *Harrison's Principle of Internal Medicine, XIII.* Isselbacher KJ, Braunwald E, Wilson GD, Martin JB, Fauci AS and Kasper DL. (eds) pp. 1618–1625. McGraw-Hill: New York.

MACLENNAN IC, KELLY K, CROCKSON RA, COOPER EH, CUZICK J AND CHAPMAN C. (1988). Results of the MRC myelomatosis trials for patients entered since 1980. *Hematol. Oncol.*, **6**, 145–158.

MEDICAL RESEARCH COUNCIL'S WORKING PARTY ON LEUKEMIA IN ADULTS. (1980). Prognostic features in the third MRC myelomatosis trial. *Br. J. Cancer*, **42**, 831–840.

MERLINI G, WALDENSTROM JG AND JAYAKAR SD. (1980). A new improved clinical staging system for multiple myeloma based on the analysis of 123 treated patients. *Blood*, **55**, 1011–1019.

MORO MS, PORTERO JA, GASCON A, HERNANDEZ JM, ORTEGA F, JIMENEZ R, GUERRAS L, MARTINEZ M, CASANOVA F, SANZ MA, GONZALES MZ AND SAN MIGUEL JF. (1992). Utility of the examination of plasma cell morphology in the study of multiple myeloma. *Sangre*, **37**, 175–179.

NIESVZKY R, SIEGEL D AND MICHAELI J. (1993). Biology and treatment of multiple myeloma. *Blood Rev.*, **7**, 24–33.

PASQUALETTI P, CASALE R, COLLACIANI A, ABRUZZO BP AND COLANTUONO D. (1990). Multiple myeloma: relationship between survival and cellular morphology. *Am. J. Heamtol.*, **33**, 145–147.

PAULE B, QUILLARD J, BISSON M, KAHN MF AND MASSIAS P. (1988). Prognostic significance of plasma cell morphology in multiple myeloma. *Nouv. Rev. Fr. Hematol.*, **30**, 209–212.

PAVLOVSKY S, CORRADO C, SANTARELLI MT, SASLAVSKY J, CAVAGNARO F, PALAU M, DE TAZANOS-PINTO M, HUBERMAN A AND LEIN JM. (1988). An update of two randomized trials in previously untreated multiple myeloma comparing melphalan and prednisone versus three- and five-drug combination: an Argentine group for the treatment of acute leukemia study. *J. Clin. Oncol.*, **6**, 769–775.

PEEST D, COLDEWEY R, DEICHER H, SAILER M, VYKOUPIL C, LEO R, GEORGII A, KAROW J, HOEPPNER E, DIEHL V, ESSERS U, GAMM H, GLÜCK S, GÖRG K, GRAMATZKI M, HAUSWALDT C, HEILMANN E, HIEMEYER V, KEYSERLINGK HJ, KINDLER A, OERTEL J, PLANKER M, REINOLD HM, SCHÄFER E, SCHUMACHER K, TIRIER C, URBANITZ D, SUCHY BR AND WÄHRISCH J. (1993). Prognostic value of clinical, laboratory and histological characteristics in multiple myeloma: improved definition of risk groups. *Eur. J. Cancer*, **29**, 978–983.

PILARSKI LM, MANT MJ AND RUETHER BA. (1985). Pre-B cells in peripheral blood of multiple myeloma patients. *Blood*, **66**, 416–419.

RICCARDI A, GOBBI PG, UCCI G, BERTOLONI D, LUONI R, RUTIGLIANO L AND ASCARI E. (1991). Changing clinical presentation of multiple myeloma. *Eur. J. Cancer*, **27**, 1401–1405.

RICCARDI A, MERLINI GP, MONTECUCCO CM, DANOVA M, UCCI G, CASSANO E AND ASCARI E. (1986). Peptichemio, vincristine and prednisone versus melphalan and prednisone as induction therapy in multiple myeloma. *Eur. J. Cancer Clin. Oncol.*, **22**, 787–791.

SAN MIGUEL JF, MORO MJ AND GONZALEZ M. (1987). Plasmablastic multiple myeloma: an immunological different subtype. (letter). *Br. J. Haematol.*, **66**, 275.

SUKPANICHNANT S., COUSAR JB, LEELASIRI A, GRABER SE, GREER JP AND COLLINS RD. (1994). Diagnostic criteria and histologic grading in multiple myeloma: histologic and immunohistologic analysis of 176 cases with clinical correlation. *Hum. Pathol.*, **25**, 308–318.

TRIBALTO M, AMADORI S AND CANTONETTI M. (1985). Treatment of multiple myeloma: a randomized trial of three different regimens. *Leuk. Res.*, **9**, 1043–1049.


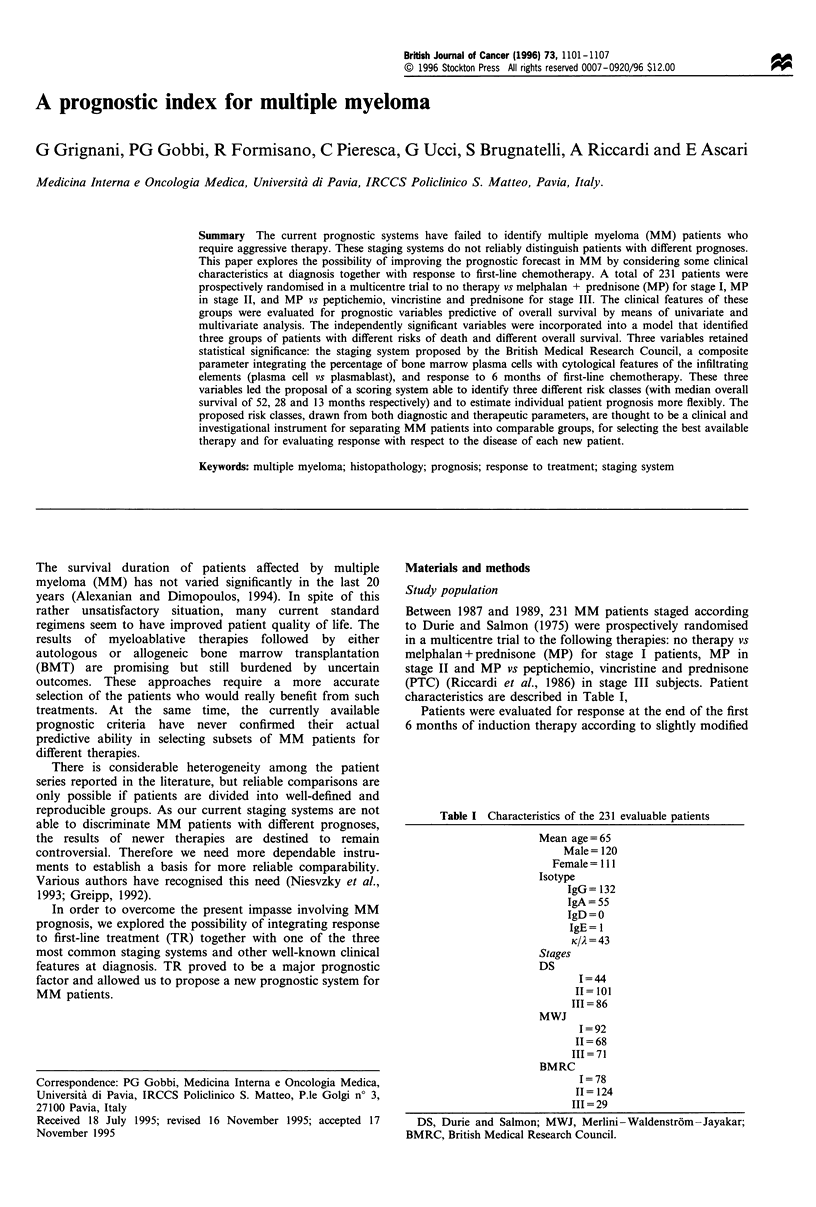

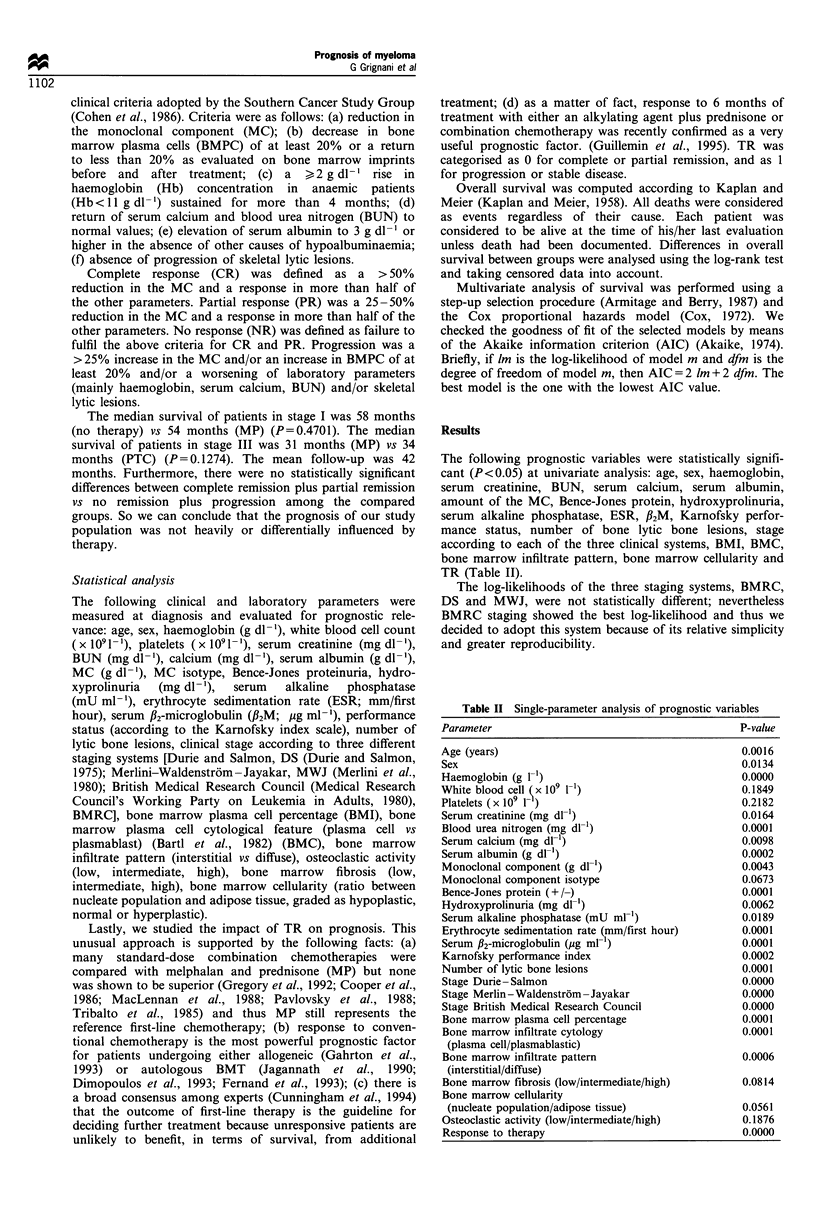

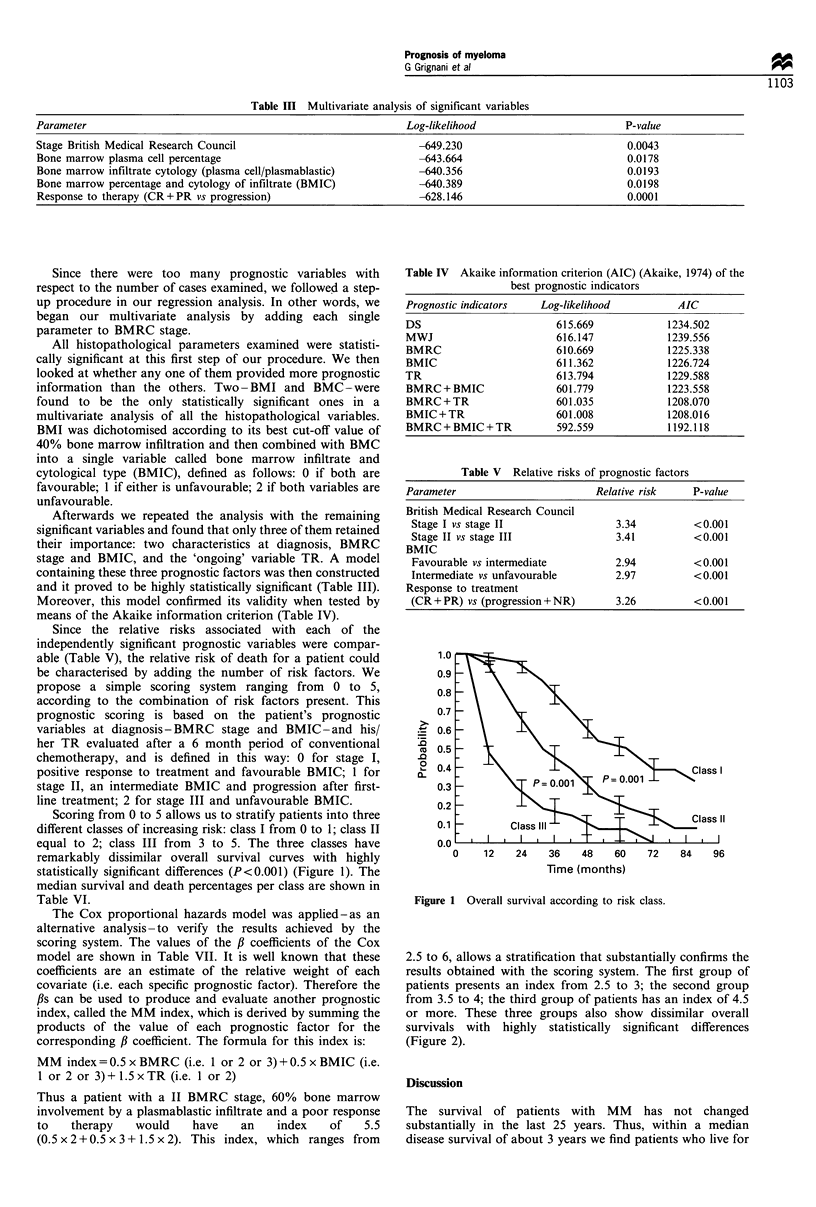

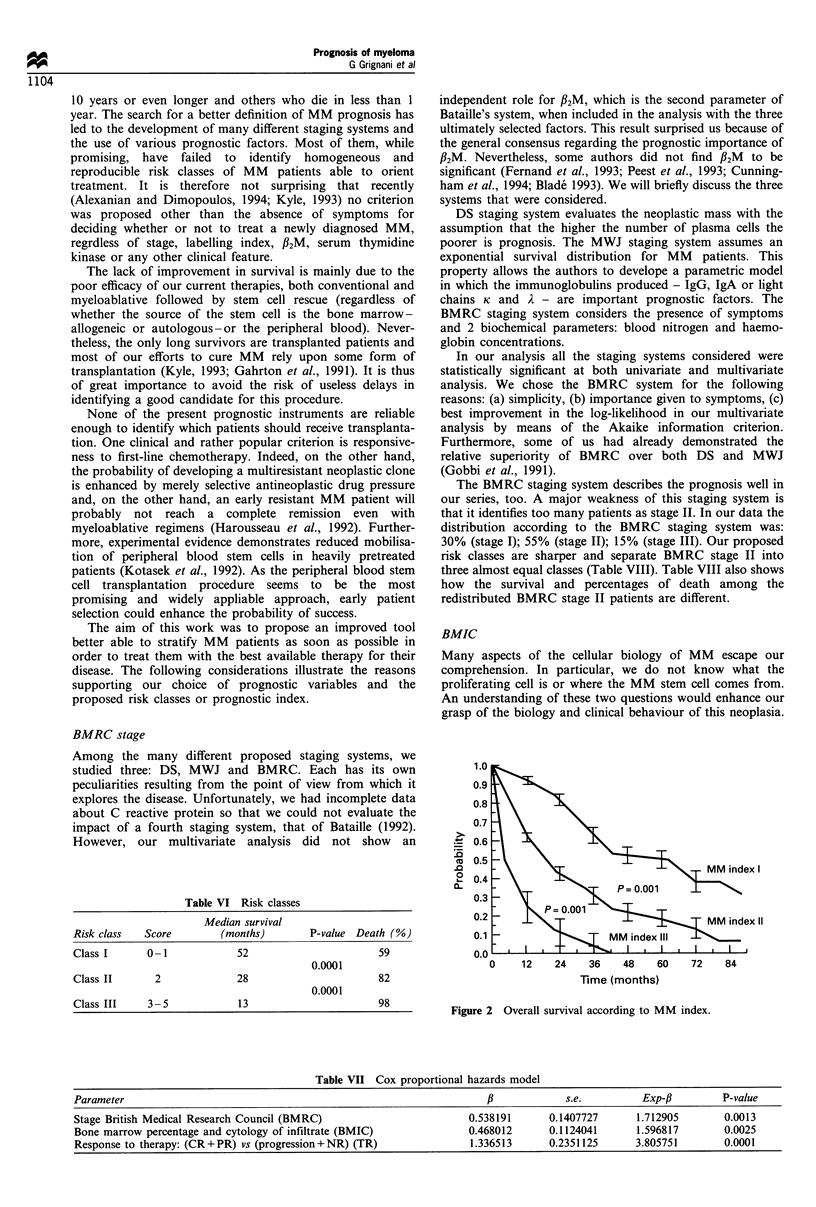

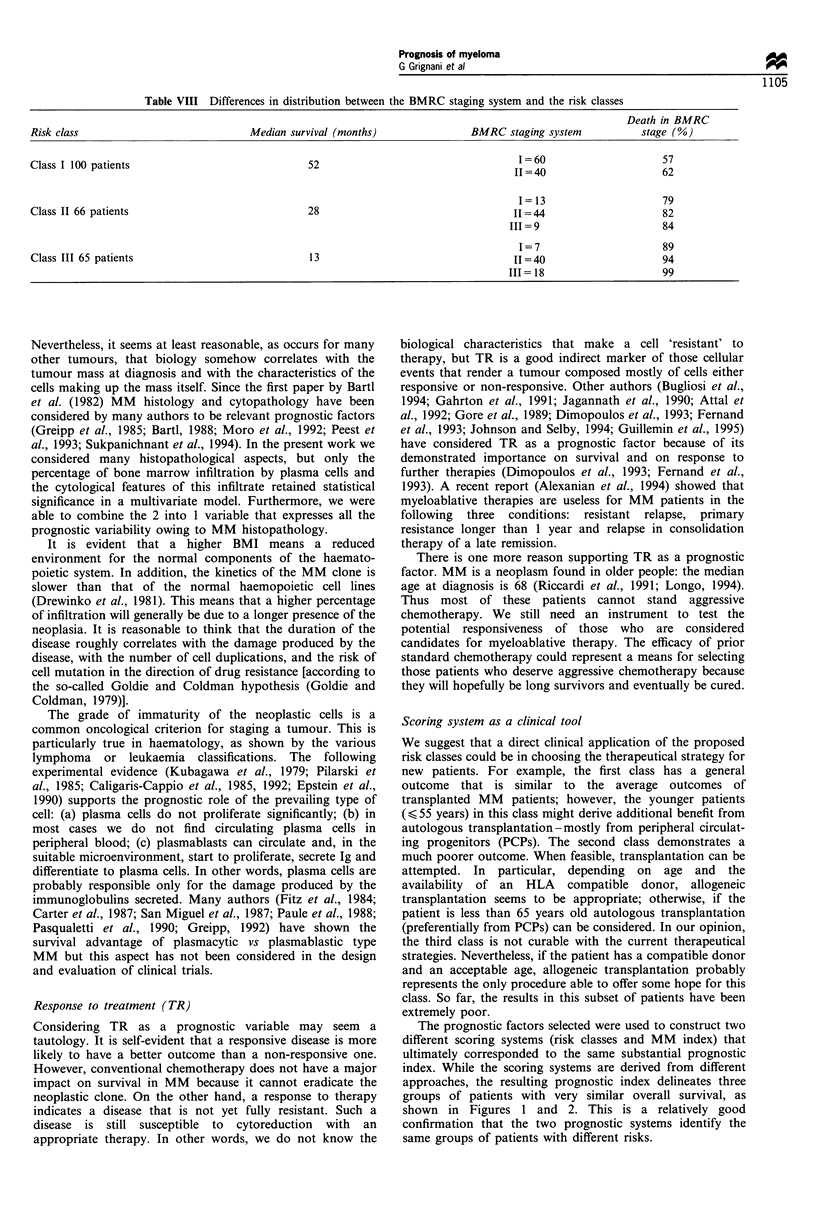

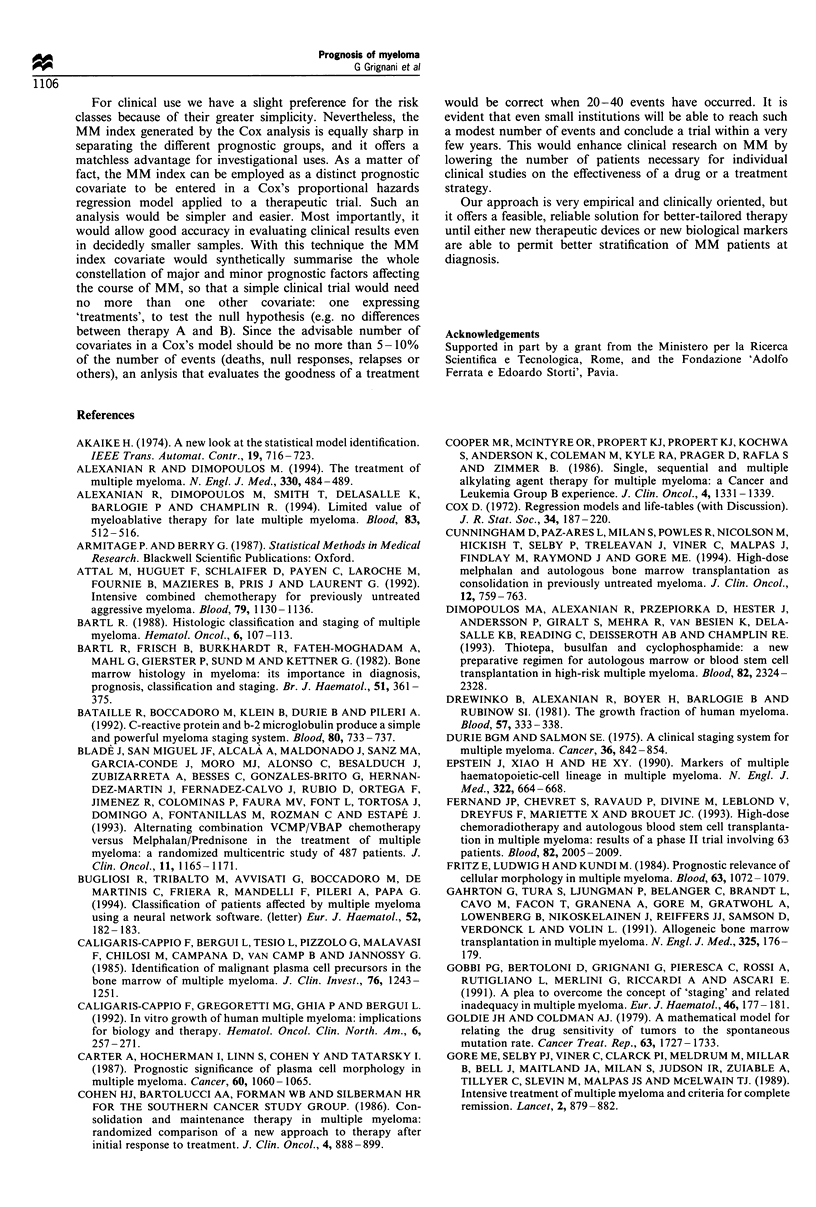

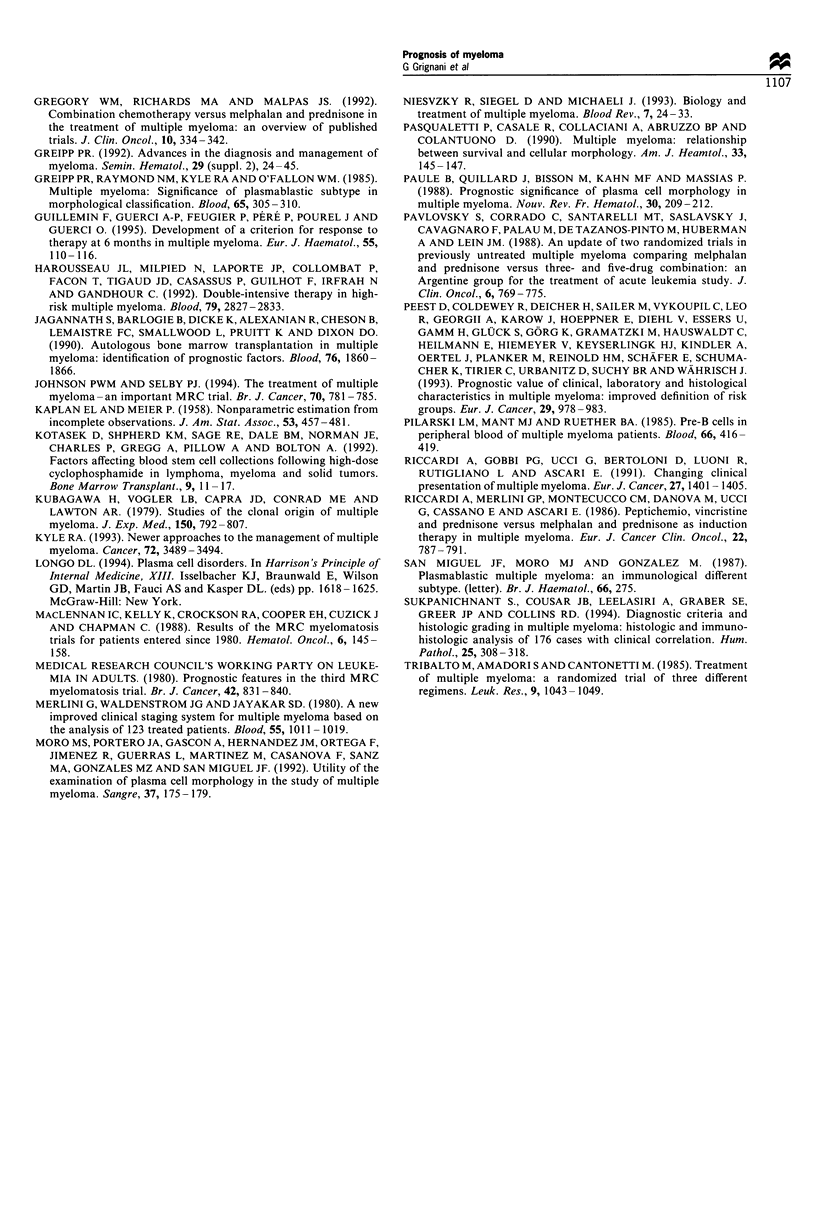


## References

[OCR_00788] Alexanian R., Dimopoulos M., Smith T., Delasalle K., Barlogie B., Champlin R. (1994). Limited value of myeloablative therapy for late multiple myeloma.. Blood.

[OCR_00784] Alexanian R., Dimopoulos M. (1994). The treatment of multiple myeloma.. N Engl J Med.

[OCR_00801] Attal M., Huguet F., Schlaifer D., Payen C., Laroche M., Fournie B., Mazieres B., Pris J., Laurent G. (1992). Intensive combined therapy for previously untreated aggressive myeloma.. Blood.

[OCR_00811] Bartl R., Frisch B., Burkhardt R., Fateh-Moghadam A., Mahl G., Gierster P., Sund M., Kettner G. (1982). Bone marrow histology in myeloma: its importance in diagnosis, prognosis, classification and staging.. Br J Haematol.

[OCR_00804] Bartl R. (1988). Histologic classification and staging of multiple myeloma.. Hematol Oncol.

[OCR_00817] Bataille R., Boccadoro M., Klein B., Durie B., Pileri A. (1992). C-reactive protein and beta-2 microglobulin produce a simple and powerful myeloma staging system.. Blood.

[OCR_00832] Bugliosi R., Tribalto M., Avvisati G., Boccardoro M., de Martinis C., Friera R., Mandelli F., Pileri A., Papa G. (1994). Classification of patients affected by multiple myeloma using a neural network software.. Eur J Haematol.

[OCR_00841] Caligaris-Cappio F., Bergui L., Tesio L., Pizzolo G., Malavasi F., Chilosi M., Campana D., van Camp B., Janossy G. (1985). Identification of malignant plasma cell precursors in the bone marrow of multiple myeloma.. J Clin Invest.

[OCR_00848] Caligaris-Cappio F., Gregoretti M. G., Ghia P., Bergui L. (1992). In vitro growth of human multiple myeloma: implications for biology and therapy.. Hematol Oncol Clin North Am.

[OCR_00854] Carter A., Hocherman I., Linn S., Cohen Y., Tatarsky I. (1987). Prognostic significance of plasma cell morphology in multiple myeloma.. Cancer.

[OCR_00857] Cohen H. J., Bartolucci A. A., Forman W. B., Silberman H. R. (1986). Consolidation and maintenance therapy in multiple myeloma: randomized comparison of a new approach to therapy after initial response to treatment.. J Clin Oncol.

[OCR_00867] Cooper M. R., McIntyre O. R., Propert K. J., Kochwa S., Anderson K., Coleman M., Kyle R. A., Prager D., Rafla S., Zimmer B. (1986). Single, sequential, and multiple alkylating agent therapy for multiple myeloma: a CALGB Study.. J Clin Oncol.

[OCR_00878] Cunningham D., Paz-Ares L., Milan S., Powles R., Nicolson M., Hickish T., Selby P., Treleavan J., Viner C., Malpas J. (1994). High-dose melphalan and autologous bone marrow transplantation as consolidation in previously untreated myeloma.. J Clin Oncol.

[OCR_00883] Dimopoulos M. A., Alexanian R., Przepiorka D., Hester J., Andersson B., Giralt S., Mehra R., van Besien K., Delasalle K. B., Reading C. (1993). Thiotepa, busulfan, and cyclophosphamide: a new preparative regimen for autologous marrow or blood stem cell transplantation in high-risk multiple myeloma.. Blood.

[OCR_00892] Drewinko B., Alexanian R., Boyer H., Barlogie B., Rubinow S. I. (1981). The growth fraction of human myeloma cells.. Blood.

[OCR_00897] Durie B. G., Salmon S. E. (1975). A clinical staging system for multiple myeloma. Correlation of measured myeloma cell mass with presenting clinical features, response to treatment, and survival.. Cancer.

[OCR_00903] Epstein J., Xiao H. Q., He X. Y. (1990). Markers of multiple hematopoietic-cell lineages in multiple myeloma.. N Engl J Med.

[OCR_00908] Fermand J. P., Chevret S., Ravaud P., Divine M., Leblond V., Dreyfus F., Mariette X., Brouet J. C. (1993). High-dose chemoradiotherapy and autologous blood stem cell transplantation in multiple myeloma: results of a phase II trial involving 63 patients.. Blood.

[OCR_00915] Fritz E., Ludwig H., Kundi M. (1984). Prognostic relevance of cellular morphology in multiple myeloma.. Blood.

[OCR_00927] Gobbi P. G., Bertoloni D., Grignani G., Pieresca C., Rossi A., Rutigliano L., Merlini G., Riccardi A., Ascari E. (1991). A plea to overcome the concept of "staging" and related inadequacy in multiple myeloma.. Eur J Haematol.

[OCR_00931] Goldie J. H., Coldman A. J. (1979). A mathematic model for relating the drug sensitivity of tumors to their spontaneous mutation rate.. Cancer Treat Rep.

[OCR_00936] Gore M. E., Selby P. J., Viner C., Clark P. I., Meldrum M., Millar B., Bell J., Maitland J. A., Milan S., Judson I. R. (1989). Intensive treatment of multiple myeloma and criteria for complete remission.. Lancet.

[OCR_00948] Gregory W. M., Richards M. A., Malpas J. S. (1992). Combination chemotherapy versus melphalan and prednisolone in the treatment of multiple myeloma: an overview of published trials.. J Clin Oncol.

[OCR_00954] Greipp P. R. (1992). Advances in the diagnosis and management of myeloma.. Semin Hematol.

[OCR_00958] Greipp P. R., Raymond N. M., Kyle R. A., O'Fallon W. M. (1985). Multiple myeloma: significance of plasmablastic subtype in morphological classification.. Blood.

[OCR_00964] Guillemin F., Guerci A. P., Feugier P., Péré P., Pourel J., Guerci O. (1995). Development of a criterion for response to therapy at 6 months in multiple myeloma.. Eur J Haematol.

[OCR_00969] Harousseau J. L., Milpied N., Laporte J. P., Collombat P., Facon T., Tigaud J. D., Casassus P., Guilhot F., Ifrah N., Gandhour C. (1992). Double-intensive therapy in high-risk multiple myeloma.. Blood.

[OCR_00973] Jagannath S., Barlogie B., Dicke K., Alexanian R., Zagars G., Cheson B., Lemaistre F. C., Smallwood L., Pruitt K., Dixon D. O. (1990). Autologous bone marrow transplantation in multiple myeloma: identification of prognostic factors.. Blood.

[OCR_00980] Johnson P. W., Selby P. J. (1994). The treatment of multiple myeloma--an important MRC trial.. Br J Cancer.

[OCR_00987] Kotasek D., Shepherd K. M., Sage R. E., Dale B. M., Norman J. E., Charles P., Gregg A., Pillow A., Bolton A. (1992). Factors affecting blood stem cell collections following high-dose cyclophosphamide mobilization in lymphoma, myeloma and solid tumors.. Bone Marrow Transplant.

[OCR_00997] Kubagawa H., Vogler L. B., Capra J. D., Conrad M. E., Lawton A. R., Cooper M. D. (1979). Studies on the clonal origin of multiple myeloma. Use of individually specific (idiotype) antibodies to trace the oncogenic event to its earliest point of expression in B-cell differentiation.. J Exp Med.

[OCR_00999] Kyle R. A. (1993). Newer approaches to the management of multiple myeloma.. Cancer.

[OCR_01009] MacLennan I. C., Kelly K., Crockson R. A., Cooper E. H., Cuzick J., Chapman C. (1988). Results of the MRC myelomatosis trials for patients entered since 1980.. Hematol Oncol.

[OCR_01020] Merlini G., Waldenström J. G., Jayakar S. D. (1980). A new improved clinical staging system for multiple myeloma based on analysis of 123 treated patients.. Blood.

[OCR_01028] Moro M. J., Portero J. A., Gascón A., Hernández J. M., Ortega F., Jiménez R., Guerras L., Martínez M., Casanova F., Sanz M. A. (1992). Utilidad del examen de la morfología de la célula plasmática en el estudio del mieloma múltiple.. Sangre (Barc).

[OCR_01034] Niesvizky R., Siegel D., Michaeli J. (1993). Biology and treatment of multiple myeloma.. Blood Rev.

[OCR_01038] Pasqualetti P., Casale R., Collacciani A., Abruzzo B. P., Colantonio D. (1990). Multiple myeloma: relationship between survival and cellular morphology.. Am J Hematol.

[OCR_01042] Paule B., Quillard J., Bisson M., Kahn M. F., Massias P. (1988). Prognostic significance of plasma cell morphology in multiple myeloma.. Nouv Rev Fr Hematol.

[OCR_01054] Pavlovsky S., Corrado C., Santarelli M. T., Saslavsky J., Cavagnaro F., Palau M., de Tezanos Pinto M., Huberman A., Lein J. M. (1988). An update of two randomized trials in previously untreated multiple myeloma comparing melphalan and prednisone versus three- and five-drug combinations: an Argentine Group for the Treatment of Acute Leukemia Study.. J Clin Oncol.

[OCR_01067] Pilarski L. M., Mant M. J., Ruether B. A. (1985). Pre-B cells in peripheral blood of multiple myeloma patients.. Blood.

[OCR_01074] Riccardi A., Gobbi P. G., Ucci G., Bertoloni D., Luoni R., Rutigliano L., Ascari E. (1991). Changing clinical presentation of multiple myeloma.. Eur J Cancer.

[OCR_01079] Riccardi A., Merlini G., Montecucco C., Danova M., Ucci G., Cassano E., Ascari E. (1986). Peptichemio, vincristine and prednisone versus melphalan and prednisone as induction therapy in multiple myeloma.. Eur J Cancer Clin Oncol.

[OCR_01085] San Miguel J. F., Moro M. J., Gonzalez M. (1987). Plasmablastic multiple myeloma: an immunologically different subtype.. Br J Haematol.

[OCR_01088] Sukpanichnant S., Cousar J. B., Leelasiri A., Graber S. E., Greer J. P., Collins R. D. (1994). Diagnostic criteria and histologic grading in multiple myeloma: histologic and immunohistologic analysis of 176 cases with clinical correlation.. Hum Pathol.

[OCR_01095] Tribalto M., Amadori S., Cantonetti M., Franchi A., Papa G., Pileri A., Boccadoro M., Dammacco F., Vacca A., Centurioni R. (1985). Treatment of multiple myeloma: a randomized study of three different regimens.. Leuk Res.

